# A new Chinese crow’s feet grading scale based on the DermaTOP system

**DOI:** 10.1038/s41598-023-46356-w

**Published:** 2023-11-02

**Authors:** Yuqing Han, Chengtong Li, Rui Wang, Jiaqi Zhang, Fan Wu, Jinfeng Zhao, Shiyu Yan, Qi Liu, Yao Pan

**Affiliations:** 1 Department of Cosmetics, School of Science, Beijing Technology and Business University, Beijing, 100048 China; 2 Beijing Key Laboratory of Plant Research and Development, Beijing, 100048 China; 3 Beijing EWISH Testing Technology Co., Ltd, Beijing, 100048 China

**Keywords:** Skin manifestations, Image processing

## Abstract

Many Chinese wrinkle studies continue to use non-Chinese scales because few Chinese-based wrinkle scales have been developed. The study aims to develop a crow’s feet grading scale for Chinese individuals. We enrolled 608 healthy Chinese subjects and measured data through the DermaTOP system. We chose exploratory factor analysis (EFA) to reduce the dimensions of the data. A three-factor structure was obtained by using EFA, and it explained a cumulative total of 89.551% of the variance. A computational formula was obtained by calculating the total factor tilt scores and taking the variance contribution rate of three factors as the weight. Based on the computational formula, a grading map was designed and tested. The model validation was conducted using both subjective assessments from the expert panel and objective results from the model calculations. The results showed that our grading scale model is stable. This study developed a Chinese crow’s feet (CCF) grading scale, which included a parameter, a grading map, and literal descriptions. The CCF grading scale is a validated tool for evaluating the effects of cosmetics or specific therapies. More importantly, the CCF scale was developed based on objective data, which may inspire new ideas for wrinkle grading scale development in the future.

## Introduction

As demand for anti-wrinkle therapy increases, related research about wrinkle prevention or treatment is increasing, highlighting the need for an objective grading scale to evaluate the effectiveness of therapies^1^. Crow's feet, also known as lateral canthal lines, are characterized by laugh lines extending from the lateral canthus to the temples. The formation of crow's feet is related to various factors, such as the movement of the orbicularis oculi muscle, the loss of elasticity, the remodeling of skin structure during aging, and photoaging. In general, crow's feet are the most common facial wrinkles. The treatment of crow's feet has become one of the most common corrective treatment requirements^2,3^.

The Asian skin aging atlas has been widely used in Chinese crow’s feet (CCF) studies. This atlas was published by the L'Oréal Evaluation and Research Center and was built on the basis of Chinese and Japanese subjects^4^. Some studies used the Japanese Cosmetic Science Society (JCSS) grading map to grade wrinkles in Chinese and Japanese subjects^5,6^. The aim was to determine differences in wrinkles among Asians from different geographical regions by comparison. Furthermore, some studies applied non-Asian standards. Cao et al.^7^ chose the Investigator's Global Assessment of Lateral Canthal Line at Rest (IGA-LCL) Severity Scale to grade crow's feet before and after three treatment approaches. Alastair^8^ developed a 5-point photonumeric rating scale to objectively quantify the severity of lateral canthal lines at rest and at maximum contracture of the orbicularis oculi. Other commonly used international grading scales for crow's feet currently include the Griffiths 10-point scale^9^, the Zerweck Crow's Feet Grading 9-point scale^10^, the Merz 5-point scale^11^, and the Lemperle score^12^. One common feature of these grading scales is that they were all developed for Caucasians; however, they have all been applied in the study of crow's feet in Chinese people.

Several studies suggest that Caucasians and Asians have different skin aging features. Richard et al.^13^ compared the skin aging features of Chinese and French women and found that wrinkle onset was delayed by approximately 10 years in Chinese women compared to French women. Tsukahara et al.^6^ found variations in skin aging features among women from Japan, China, and Thailand. Importantly, most wrinkle-grading scales are created based on pictures subjectively selected by experts or dermatologists. However, human subjective judgment is subject to error, and the method may be unreliable. Therefore, we decided to use objective instruments to measure crow's feet and to grade them based on the measured objective data.

We believe that a crow’s feet grading scale for Chinese individuals is necessary because Chinese individuals represent 31.69% of the Asian population. In this study, we used the DermaTOP system to establish a CCF grading scale. In total, 608 subjects were tested, and 14 parameters were captured for each subject. We established a computational formula for crow's feet severity and photographic scales using parameters provided by the DermaTOP system. As an objective, quantitative grading scale, it can be used for daily purposes and as a clinical scale for evaluating the severity of crow's feet in Chinese individuals.

## Methods

### Instrument

A DermaTOP^®^ system (Eotech, Marcoussis, France) was used to capture images of crow’s feet. The images were analyzed by the DermaTOP^®^ system software. In the DermaTOP system, the threshold value for the object area was set to 0.5 mm2, the threshold value for the number of objects was set to 100, and the threshold value for the histogram was set to 20%. The outer canthus of the subject was selected as the test area, and the shooting angle was set to 60° on both the left and right sides. The positioning system kept the test area stable during image collection. Due to the working principle of the DermaTOP system, the captured images were black and white. To compensate for this deficiency, color photographs were taken by a VISIA^®^ CR (Canfield Scientific Inc., Fairfield, NJ, USA). Color images taken by the VISIA^®^ CR were used to create the grading map.

### Subjects

Six hundred and eight healthy Chinese subjects with Fitzpatrick skin type III or IV, aged 18–60 years, were recruited from Beijing, China. This study was conducted according to the principles of the Declaration of Helsinki and Good Clinical Practices (GCP) regulations. The study protocol was approved by the Shanghai Ethics Committee for Clinical Research. All procedures involved in the study were explained in detail, and written informed consent was obtained from all 608 subjects. Subjects were selected after ensuring that they were not taking any medication influencing their skin condition. Subjects were excluded if they were pregnant, had a recent medical procedure in the test area, or were participating in another clinical study. All subjects refrained from applying products to their faces. Data from 428 of these subjects were used to build the model, while data from 180 subjects were used to validate it.

### Study design

To avoid environmental influences, the subjects were acclimated in the examination room at a temperature of 22 ± 1 °C and relative humidity of 50 ± 5% for 30 min after washing their faces. The DermaTOP system and VISIA^®^ CR were used to capture crow’s feet images. Right and left oblique views were captured using a standard procedure. VISIA^®^ CR using s1 natural light source mode. DermaTOP images were analyzed using its proprietary analysis software and exported as data for model establishment. VISIA^®^ CR output images were used as references without any additional data analysis. In the validation phase, we compared the subjective grading results of an expert panel with the results obtained from the model calculations. The panel consisted of four dermatologist-trained researchers, each of whom has been in practice for more than 5 years. The images taken by the VISIA^®^ CR for 180 subjects were displayed on a PC and initially graded by the expert assessment panel. The subjective assessment results of the expert panel were then analyzed against the CCF parameters using receiver operating characteristic (ROC) curve analysis.

### Statistical analysis

The DermaTOP system analysis software was used to analyze the images. The data were analyzed by using IBM SPSS Statistics for Windows, version 22.0 (Armonk, NY: IBM Corp. Released 2013). Exploratory factor analysis (EFA) was evaluated using the Kaiser‒Meyer‒Olkin (KMO) test and Bartlett's test of sphericity. A principal component analysis (PCA) correlation matrix was used to extract the factors. Varimax rotation was used to calculate the rotated component matrix. The significance level was set at α = 0.05. The ROC curves were analyzed and plotted using GraphPad Prism 9.

## Results

### Original measurement parameters

Images were taken of 608 subjects (80 males and 528 females). The demographic characteristics of the study subjects are shown in Table 1. The mean age of the subjects was 40.79 ± 11.85 years. Most of the subjects were aged 41–50 years, followed by 51–60 years.

**Table 1:** Description of the population.

Age group	Age range	Average age	Total
1	18–30	23.67 ± 1.95	116
2	31–40	35.05 ± 2.71	140
3	41–50	46.34 ± 3.87	206
4	51–60	52.09 ± 2.72	146
Total	18–60	40.79 ± 11.85	608

Fourteen original wrinkle parameters were obtained from each image. The descriptive analysis of the test results and the item numbers are shown in Table 2. The LR values were all greater than 1 and ranged from 1.0065 to 1.1720, indicating that the real length of the wrinkles or textures in the test area was greater than the length of the lines in the test area, and the skin surface was not absolutely smooth. The volume sum, circumference sum and area sum values were positive, and the max depth average and mean depth average values were negative. All parameters were within normal ranges. The intraclass correlation coefficient (ICC) for all items was greater than 0.9, indicated excellent reproducibility of the data.

**Table 2:** Information on 14 parameters extracted from the images.

Item	Parameter measured	Parameter description	N	Minimum	Maximum	Mean	SD	ICC
B1	LR	The length ratio	428	1.0065	1.1720	1.0287	0.0197	0.9648
B2	Ra	Average roughness	428	0.0116	0.0828	0.0249	0.0082	0.9347
B3	Rz	Mean maximum height deviation	428	0.0498	0.2940	0.0999	0.0320	0.9135
B4	Rt	2D maximum height deviation	428	0.0891	0.5492	0.1861	0.0637	0.9905
B5	SR	The area ratio	428	1.0157	1.4753	1.0562	0.0419	0.9686
B6	SQ	Quadratic mean deviation	428	0.0150	0.1056	0.0329	0.0110	0.9923
B7	ST	3D maximum height deviation	428	0.1960	2.6819	0.4089	0.1939	0.9711
B8	SA	3D linear arithmetic mean	428	0.0115	0.0862	0.0250	0.0081	0.9606
B9	Stm	Maximum height deviation mean	428	0.1256	0.7866	0.2460	0.0794	0.9481
B10	Volume sum	Volume of the depression in the skin	428	0	2.5910	0.1059	0.2665	0.9983
B11	Circumference sum	Circumference of the depression in the skin	428	0	178.5700	14.9387	26.6017	0.9253
B12	Area sum	Area of the depression in the skin from skin	428	0	29.8550	1.9639	3.8037	0.9344
B13	Max depth average	Max depth of the depression in the skin	428	− 0.4040	0	− 0.0550	0.0724	0.9866
B14	Mean depth average	Average depth of the depression in the skin	428	− 0.1020	0	− 0.0172	0.0219	0.9234

### CCF parameters

Pairwise correlations showed a high positive correlation between most parameters (Table 3). Usually, |r|< 0.2 indicates a weak linear correlation between two variables. Ten of the 105 pairs showed significant differences of *p* ≥ 0.01: B5 and B10, B5 and B11, B5 and B12, B5 and B13, B5 and B24, B9 and B10, B9 and B11, B9 and B12, B9 and B13, and B9 and B14. The remaining pairs showed significant differences of *p* < 0.01, which meant that these original parameters were suitable for the factor analysis^14,15^.

**Table 3:** Correlation matrix.

	B1	B2	B3	B4	B5	B6	B7	B8	B9	B10	B11	B12	B13	B14
B1	1													
B2	0.872**	1												
B3	0.891**	0.988**	1											
B4	0.861**	0.970**	0.983**	1										
B5	0.437**	0.427**	0.391**	0.375**	1									
B6	0.431**	0.505**	0.483**	0.465**	0.843**	1								
B7	0.279**	0.329**	0.322**	0.317**	0.712**	0.814**	1							
B8	0.441**	0.513**	0.485**	0.465**	0.874**	0.993**	0.779**	1						
B9	0.410**	0.445**	0.424**	0.409**	0.913**	0.939**	0.839**	0.940**	1					
B10	0.162**	0.287**	0.280**	0.290**	0.009	0.334**	0.298**	0.301**	0.159	1				
B11	0.155**	0.281**	0.273**	0.282**	0.014	0.318**	0.271**	0.285**	0.136	0.887**	1			
B12	0.154**	0.280**	0.272**	0.282**	0.012	0.319**	0.277**	0.286**	0.139	0.940**	0.984**	1		
B13	0.172**	0.292**	0.285**	0.295**	0.054	0.344**	0.320**	0.314**	0.188	0.569**	0.422**	0.463**	1	
B14	0.176**	0.297**	0.288**	0.298**	0.065	0.349**	0.320**	0.321**	0.196	0.538**	0.376**	0.417**	0.939 **	1

“**” indicates a significant difference, *p* < 0.01 (two-tailed).

Whether the original index satisfied the precondition for EFA was judged by Bartlett's test of sphericity and the KMO test of the original variables^14,15^. In principle, a KMO greater than 0.6 and a P value less than 0.05 are required to perform EFA. As shown in Table 4, the chi-square value of Bartlett's test of sphericity was 13,412.740 (*p* < 0.01), and the KMO value was 0.791, which indicated that the 14 original parameters were suitable for EFA.

**Table 4:** KMO and Bartlett’s test.

Kaiser–Meyer–Olkin measure of sampling adequacy		0.791
Bartlett’s test of sphericity	Approx. Chi-square	13,412.740
	df	91
	Sig	0.000

Based on the correlation coefficient matrix of the 14 original parameters, we extracted factors by PCA with a characteristic root > 1. The results are summarized in Table 5. A total of 89.551% of the total variance of the original parameters was explained by three factors. In general, the information loss of the original parameters was low, and the EFA results obtained an ideal effect.

**Table 5:** Total variance explained.

Component	Initial Eigenvalues			Extraction sums of squared loadings			Rotation sums of squared loadings		
	Total	% of Variance	Cumulative %	Total	% of Variance	Cumulative %	Total	% of Variance	Cumulative %
1	7.014	50.097	50.097	7.014	50.097	50.097	4.411	31.506	31.506
2	3.293	23.519	73.616	3.293	23.519	73.616	4.270	30.501	62.007
3	2.231	15.934	89.551	2.231	15.934	89.551	3.856	27.543	89.551
4	0.705	5.034	94.585						
5	0.269	1.923	96.508						
6	0.196	1.402	97.910						
7	0.102	0.731	98.640						
8	0.094	0.675	99.315						
9	0.043	0.307	99.622						
10	0.026	0.184	99.806						
11	0.014	0.101	99.907						
12	0.007	0.052	99.960						
13	0.004	0.027	99.987						
14	0.002	0.013	100.000						

Extraction method: Principal component analysis.

The component score coefficient was estimated by the regression method. As detailed in Table 6, the extraction method adopted PCA, and the rotation scheme used KMO. The factor loading of each parameter was > 0.8, so no parameters were removed. Factor 1 included B9 (Stm), B5 (SR), B8 (SA), B6 (SQ), and B7 (ST); Factor 2 included B12 (area sum), B11 (circumference sum), B10 (volume sum), B13 (max depth average), and B14 (mean depth average); and Factor 3 included B3 (Rz), B4 (Rt), B2 (Ra), and B1 (LR). Based on the original parameters included in each factor, we named Factor 1 “three-dimensional data of wrinkles”, Factor 2 “aggregate data of wrinkles”, and Factor 3 “two-dimensional data of wrinkles”.

**Table 6:** Rotated component matrix.

	Component		
	1	2	3
B9	0.956		
B5	0.917		
B8	0.915		
B6	0.907		
B7	0.845		
B12		0.951	
B11		0.95	
B10		0.898	
B13		− 0.844	
B14		− 0.833	
B3			0.958
B4			0.948
B2			0.947
B1			0.902

Extraction method: Principal component analysis. Rotation method: Varimax with Kaiser normalization. Rotation converged in 5 iterations.

Table 7 shows the results of the coefficient matrix of the component score. Then, the factor score functions were obtained as follows:

$$ \begin{aligned} {\text{F}}_{1} & = - 0.052*{\text{B}}1 - 0.056*{\text{B}}2 - 0.068*{\text{B}}3 - 0.073*{\text{B}}4 + 0.25*{\text{B}}5 \\ & \quad + \;0.222*{\text{B}}6 + 0.226*{\text{B}}7 + 0.227*{\text{B}}8 + 0.2540.44*{\text{B}}9 \\ & \quad - \;0.008*{\text{B}}10 - 0.043*{\text{B}}11 - 0.036*{\text{B}}12 + 0.033*{\text{B}}13 + 0.036*{\text{B}}14 \\ {\text{F}}_{2} & = - 0.037*{\text{B}}1 - 0.020*{\text{B}}2 - 0.013*{\text{B}}3 - 0.008*{\text{B}}4 - 0.085*{\text{B}}5 \\ & \quad + \;0.003*{\text{B}}6 + 0.013*{\text{B}}7 - 0.016*{\text{B}}8 - 0.044*{\text{B}}9 + 0.224*{\text{B}}10 \\ & \quad + \;0.242*{\text{B}}11 + 0.242*{\text{B}}12 - 0.204*{\text{B}}13 - 0.203*{\text{B}}14 \\ {\text{F}}_{3} & = 0.269*{\text{B}}1 + 0.278*{\text{B}}2 + 0.285*{\text{B}}3 + 0.283*{\text{B}}4 - 0.040*{\text{B}}5 \\ & \quad - \;0.042*{\text{B}}6 - 0.090*{\text{B}}7 - 0.036*{\text{B}}8 - 0.057*{\text{B}}9 - 0.049*{\text{B}}10 \\ & \quad - \;0.028*{\text{B}}11 - 0.037*{\text{B}}12 - 0.016*{\text{B}}13 - 0.015*{\text{B}}14. \\ \end{aligned} $$

**Table 7:** Component score coefficient matrix.

	Component		
	1	2	3
B1	− 0.052	− 0.037	0.269
B2	− 0.056	− 0.020	0.278
B3	− 0.068	− 0.013	0.285
B4	− 0.073	− 0.008	0.283
B5	0.250	− 0.085	− 0.040
B6	0.222	0.003	− 0.042
B7	0.226	0.013	− 0.090
B8	0.227	− 0.016	− 0.036
B9	0.254	− 0.044	− 0.057
B10	− 0.008	0.224	− 0.049
B11	− 0.043	0.242	− 0.028
B12	− 0.036	0.242	− 0.037
B13	0.033	− 0.204	− 0.016
B14	0.036	− 0.203	− 0.015

Extraction method: principal component analysis. Rotation method: Varimax with Kaiser normalization. Rotation converged in 5 iterations.

We obtained the following computational formula by calculating the total factor tilt scores and taking the variance contribution rate of three factors as the weight:

$$ {\text{F}} = 0.31506{\text{F}}_{1} + 0.30501{\text{F}}_{2} + 0.27543{\text{F}}_{3} . $$

The F value was used as the aggregative indicator of the CCF grading scale, which was named the CCF index.

### CCF grading map

We also developed a CCF grading map. Table 8 shows the results and the statistical description. The CCF parameter ranged between 0.115318 and 11.276754. The larger the CCF parameter was, the more serious the crow's feet were. The CCF parameters obtained from the synthesis are arranged in ascending order and then divided into 12 groups with equal spacing between each group.

**Table 8:** Descriptive statistics of the CCF parameter results.

	N	Range	Minimum	Maximum	Mean	SD
CCF parameters	428	11.161437	0.115318	11.276754	1.04217648	1.616725145
N of valid	428					

**Figure 1:**
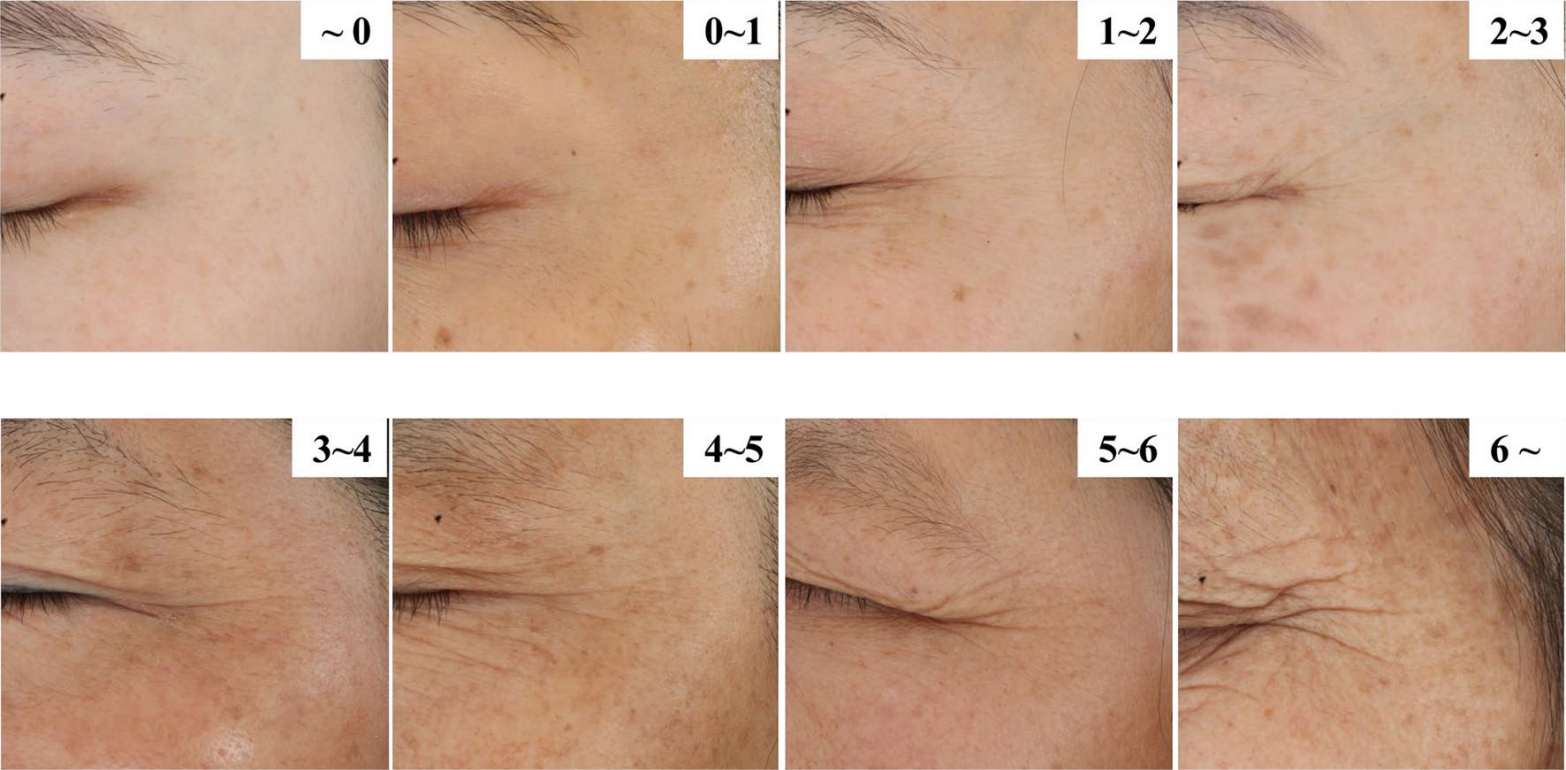
The CCF grading map.

The group distance of each group is calculated by the extreme difference value and dividing it by the number of groups. The group limit value is then determined based on this calculation. Using the group limit value, the corresponding images are retrieved, resulting in a total of 13 images. Then, the photographs were screened by an expert assessment panel. Finally, eight photographs with discernable differences in visual assessment became the standard photographs for the CCF grading map. Based on the practicability of the CCF grading map, each photograph's name was set from n to n + 1. Once the assessment result was between two photographs, the crow's feet could be graded. The grading map is shown in Fig. 1.

The CCF parameters and literal descriptions of the different photographs in the CCF grading map are summarized in Table 9. In addition, the range of the CCF parameter corresponding to the CCF grading scale is summarized in Table 10.

**Table 9:** The CCF parameters and literal descriptions of different images in the CCF grading map.

Image name	CCF parameters	Literal descriptions
~ 0	0.1153	The wrinkles are invisible
0–1	0.5767	The wrinkles are hardly visible
1–2	1.9562	The wrinkles are visible, but small and clustered
2–3	3.6065	The wrinkles are visible
3–4	4.6173	The wrinkles are visible and mild
4–5	6.657	The wrinkles are visible and moderate
5–6	6.9143	The wrinkles are visible, deep and clear
6–	8.6717	The wrinkles are visible, deep, clear and thick

**Table 10:** The range of CCF parameters in the CCF grading scale.

Level	Range of CCF parameters
0	< 0.5767
1	0.5767–1.9562
2	1.9562–3.6065
3	3.6065–4.6173
4	4.6173–6.6570
5	6.6570–6.9143
6	6.9143–8.6717

### Confirmatory experiment

We recruited 180 subjects for model validation. The CCF parameters were initially calculated for the 180 subjects using the model and then objectively graded based on the CCF ranges specified in Table 9. Afterward, the images of the 180 subjects were displayed on a PC, and each image was subjectively graded twice by the four trained researchers from the expert panel. The two subjective ratings were performed 1 month apart. The kappa coefficients of the two subjective ratings of the images generated by each researcher were all greater than 0.75, indicating a high level of agreement between the two assessments. Additionally, the kappa coefficient among the four researchers was 0.908, indicating strong agreement among the assessment panels. The above results indicated that the subjective assessment panel can ensure the accuracy of the wrinkle grading assessment results (Table 11).

**Table 11:** Coherence of the assessment panels.

Item	Kappa	*p*
Two evaluations by researcher 1	0.875	< 0.01
Two evaluations by researcher 2	0.847	< 0.01
Two evaluations by researcher 3	0.756	< 0.01
Two evaluations by researcher 4	0.750	< 0.01
Among the 4 researchers	0.908	< 0.01

**Figure 2:**
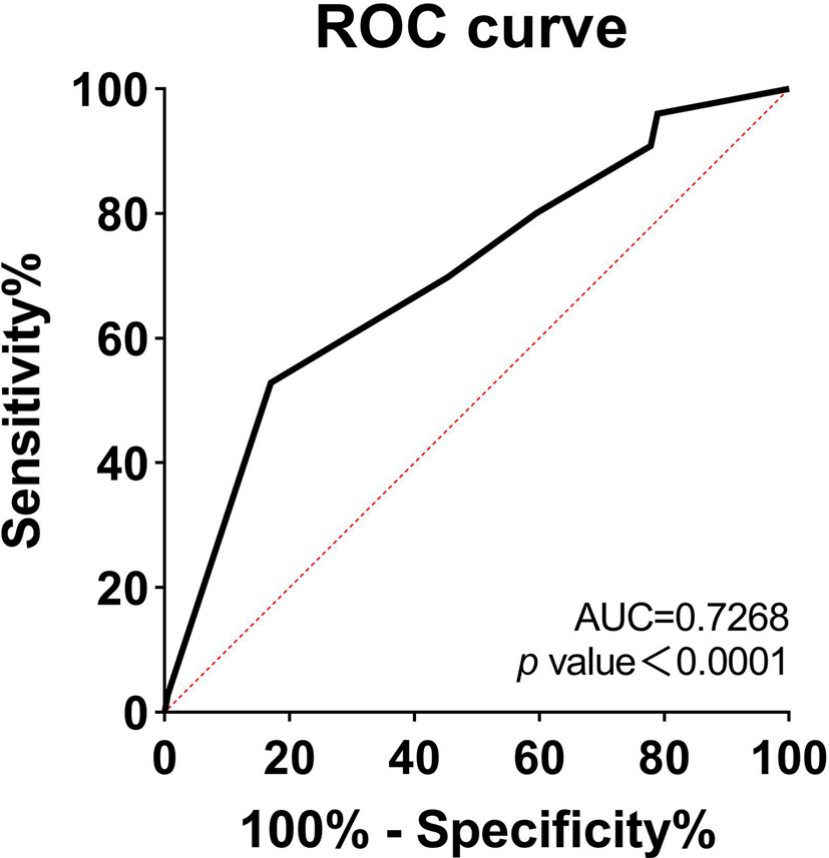
Characteristic diagram of ROC curves.

ROC curves were plotted and analyzed using the mean of the subjective assessment results from the four trained researchers and the subjects' CCF parameters (Fig. 2). The area under the ROC curve (AUC) was calculated to be 0.7268, with a *p* value < 0.0001. The confirmatory experimental results showed that the CCF grading scale model is stable.

## Discussion

Many studies have found that Caucasians and Asians have different skin aging features^13^. There are even slight differences in skin aging features between Asian countries^6^. Most Chinese studies on aging evaluation in recent years have been relegated to predicting age using wrinkle-grading scales developed by Caucasians. The treatment of crow’s feet has become one of the most common corrective treatment requirements^2^. Therefore, an objective grading scale is needed to evaluate treatment effectiveness. Based on 3D parameters, we developed and tested a CCF grading scale with an objective formula, a grading map, and literal descriptions.

For most grading scales, it is necessary to know how and why their scores were developed or which photographs were chosen^1^. Therefore, we need to clearly show the development process. We enrolled 608 healthy Chinese subjects. The demographic characteristics of the subjects are shown in Table 1. Quantifying parameters can provide much detailed information that is not visible to the naked eye. However, 2D imaging can only measure the length and width of wrinkles, not height. Imaging is also severely limited by the environment, such as light intensity and color temperature. In addition, 2D imaging has difficulty achieving accurate positioning. The DermaTOP system measures the three-dimensional parameters of wrinkles, allows for precise positioning and is not affected by the environment. Lagarde et al.^16^ ran three experiments to evaluate the accuracy, repeatability, and reproducibility of the DermaTOP system. They confirmed the suitability of the DermaTOP system for the evaluation of the micro- and macrotopography of the skin. This system has been validated for the quantitative evaluation of facial skin features^17,18^. Eventually, fourteen parameters related to crow's feet were measured through the DermaTOP system. Information on these 14 parameters is shown in Table 2. This is the first time that the DermaTOP system has been used in the study of wrinkle grading.

EFA is widely used in developing and verifying scales^19^ and is also used for mathematical modeling in medical research^20^. In this paper, we innovatively applied EFA to wrinkle grading. Tables 3 and 4 show that the 14 original parameters were suitable for EFA. Table 5 shows that the three factors explained 89.551% of the total variance of the original parameters. Overall, the information loss of the original parameters was low, and the effect of EFA was ideal. After calculating the factor loading (Table 6) and factor score (Table 7), we obtained the computational formula F = 0.31506F_1_ + 0.30501F_2_ + 0.27543F_3_. We named this the CCF parameter.

An objective evaluation is more reliable than a subjective visual evaluation. The CCF parameter can objectively evaluate crow's feet. However, given that the DermaTOP system may not be widely used, we decided to develop a CCF grading map. Griffiths et al.^21^ and Marks et al.^22^ conducted comparative experiments with descriptive and photographic grading scales. The results demonstrated that photographic grading scales are much more sensitive than descriptive scales. To summarize, we developed a grading map with photographs, descriptions, and CCF parameters. We used the computational formula to calculate the CCF parameter of all images (Table 8) and divided them into 12 groups of equal width. We determined the group limits by calculating the interval range and number of distinct categories for each group. The expert assessment panel selected eight photographs according to the group limits, which became the standard photographs of the CCF grading map (Fig. 1). Then, the expert assessment panel named and described each photograph. The photograph names, descriptions, and CCF parameters are shown in Table 9. The range of the CCF parameter corresponding to the CCF grading map is summarized in Table 10. In light of the grading map's actual use, we named the eight photographs 0–1, 1–2 and so on so that the user could quickly grade the subject's crow's feet. If the crow's feet are between photographs 0–1 and 1–2, they can be graded as level 1.

Validity plays a vital role in wrinkle-grading studies. A systematic review of wrinkle-grading scales indicated that the validity of only a minority of published scales is supported by adequate scientific evidence^1^. In the confirmatory experiment, we asked four trained researchers to use the CCF grading map to grade 180 images. The results showed a consistent probability between the subjective assessment of the expert assessment panel and the CCF parameters. In general, the higher the AUC, the more accurate the model is. The AUC of the ROC curve was 0.7268, indicating that the CCF grading scale model is stable. It also proves that the CCF parameter and the CCF grading map are highly consistent. The experimental results fully proved the validity of the CCF grading scale.

Tan et al.^23^ developed and validated a visual assessment for the severity of lacrimal groove wrinkles in Chinese females. Their scale was developed based on the hierarchical description of the Lemperle photometric scale and the evaluation description of lacrimal groove depression in Francois’ AIRS scale. Three evaluators selected the corresponding photos according to the textual descriptions of the above scales and composed the graded scales. Finally, the photos of subjects with high consensus were used as the legend of the clinical evaluation scale. However, the Lemperle photometric scale and Francois’ AIRS scale were developed based on Caucasians. We do not know if these scales are applicable to Chinese women. In our paper, we used objective instruments to directly capture wrinkle parameters in Chinese people and developed the CCF parameter and a CCF map. This scale development method effectively avoided the errors caused by ethnic differences. Therefore, the CCF scale will be more suitable for Chinese people. Zhang et al.^24^ established the lateral canthus (both static and dynamic), glabellar, forehead (both static and dynamic), and nasolabial fold grading scales and determined their validity. However, two dermatologists classified photos by midpoint depth between the wrinkles. Only after the classification was completed did they quantify wrinkles using objective instruments. Instead, we quantified wrinkles with objective instruments and then relied on wrinkle data for photograph selection. Therefore, the development process of the CCF scale was more objective.

Based on 3D parameters, we developed and tested a CCF grading scale with an objective formula, a grading map and literal descriptions. We showed the development process and the test process. The CCF grading scale is transparent and effective. In terms of innovation, this is the first time that the DermaTOP system has been used in the study of wrinkle grading. This is also the first time that EFA was applied to a wrinkle-grading study. However, we should have considered that the DermaTOP system may not be widely used when we chose the measuring instrument. This may result in a poor application of the CCF parameter since the original parameters need to be measured with the DermaTOP system.

## Conclusion

In this paper, we developed and tested a CCF grading scale. It includes a CCF parameter, a CCF grading map, and literal descriptions. The CCF parameter is a method for the quantitative evaluation of crow's feet. In comparison, the CCF grading map is suitable for subjective grading. They can be used together or alone. The confirmatory experiment showed that the CCF grading scale was valid and valuable. The CCF grading scale is a validated tool for evaluating the effects of cosmetics or specific therapies.

### Statement

This study was conducted according to the principles of the Declaration of Helsinki and Good Clinical Practices (GCP) regulations. The study protocol was approved by the Shanghai Ethics Committee for Clinical Research. All procedures involved in the study were explained in detail, and written informed consent was obtained from all 428 subjects.

## Data Availability

The datasets used and analyzed during the current study are available from the corresponding author upon reasonable request.
